# Initial programme theory developing for interprofessional case discussions (InCaD) in acute hospital care: a realist approach

**DOI:** 10.1186/s12913-025-13865-5

**Published:** 2025-12-11

**Authors:** Julien Pöhner, Kathrin Seibert, Eva-Maria Regelmann, Henrikje Stanze

**Affiliations:** School of Social Sciences, University of Applied Sciences Bremen, Am Brill 2-4, 28195 Bremen, Germany

**Keywords:** Interprofessional case discussions, Realist methodology, Programme theory development, Context-Mechanism-Outcome (CMO) configurations, Quality improvement in healthcare

## Abstract

**Background:**

Interprofessional case discussions (InCaD) are vital for managing complex patient care in acute hospital settings. Despite their widespread use, the absence of theoretical frameworks hinders consistent implementation and evaluation. This study aims to develop an initial programme theory for InCaD using a realist methodology to identify Context-Mechanism-Outcome (CMO) configurations.

**Methods:**

This study employed a multi-method approach grounded in the realist methodology following a prospective approach of the RAMESES II reporting standards for realist evaluations. Data were collected from three sources: internal programme documents, a systematic review (*N* = 42), and stakeholder focus group interviews (*N* = 17) involving nurses, physicians, managers and therapists. Internal programme documents provided an understanding of theoretical foundations and informed the review and interview design. The systematic review followed PRISMA 2020 guidelines and included studies on multiprofessional case discussions in inpatient settings. Focus group interviews were conducted with hospital stakeholders to explore practical insights into the implementation and impact of InCaD. Data synthesis involved generating CMO-configurations through thematic coding and iterative team discussions.

**Results:**

The analysis identified direct mechanisms, such as interprofessional reflection, structured protocols, and shared decision-making, and indirect mechanisms, such as fostering team trust and a culture of learning. Key outcomes for patients included improved care quality, satisfaction, and continuity, while staff outcomes encompassed enhanced job satisfaction, team cohesion, and workflow efficiency. Contextual factors, including leadership support and tailored adaptations, were crucial for successful implementation. Nurse leadership emerged as a significant driver of InCaD’s effectiveness.

**Conclusions:**

This study provides a theory-driven framework for systematically implementing and evaluating InCaD in clinical practice. By understanding the interactions of context, mechanisms, and outcomes, healthcare organizations can enhance patient safety, care quality, and staff well-being. Future research should validate and refine these findings in diverse healthcare settings to inform broader applications.

**Supplementary Information:**

The online version contains supplementary material available at 10.1186/s12913-025-13865-5.

**Table:** 

Text box 1. Contributions to the literature
• This study develops an initial programme theory for interprofessional case discussions (InCaD), addressing a critical gap in the theoretical foundation for their implementation and evaluation in acute hospital care.
• By employing a realist methodology, the study identifies key CMO-configurations, offering insights into how and why InCaD generates change in complex healthcare settings.
• This work highlights the importance of context-specific adaptations and the pivotal role of nurse leadership in enhancing the effectiveness and sustainability of InCaD.
• The proposed framework provides healthcare organizations with a systematic approach to implement and evaluate InCaD, contributing to improved patient safety, care quality, and staff well-being.

## Background

Extended life expectancies and demographic changes are leading to an increase in chronic diseases and in the complexity of nursing care [1, 2]. Healthcare providers are confronted with highly complex care situations that focus not only on nursing but also on medical and ethical challenges such as the care of multimorbid patients. Advances in medicine and digital technologies have contributed to this development by enabling a wider range of care scenarios [3]. The inpatient acute care sector is particularly affected by this, as patients often require intensive care and close interprofessional collaboration to ensure coordinated and effective care [4]. Managing these dynamic interactions among the various challenges in the healthcare system requires well-considered and comprehensive strategies [5]. This requires not only considering the current needs and requirements of healthcare professionals such as nurses, physicians or therapists, and patients but also finding a sustainable balance between the quality of patient care and the efficiency of the healthcare system [6]. In this context, patient-centered care with the focus on the patient and the individual’s particular health care needs and to empower patients to become active participants in their care is essential [7]. Each profession contributes differently to this aim: nurses ensure continuity and patient advocacy, physicians provide diagnosis and treatment planning, and therapists address functional and psychosocial aspects of care [8]. To achieve this goal, interprofessional collaboration and communication like collaborative decision-making for well-founded treatment decisions through the exchange of expertise or coordination of care to meet patient needs and ensure patient safety [9] are central building blocks and are considered indispensable for optimising quality of care. For this, Interprofessional case discussions (InCaD) with the content of collaboration and communication can be helpful. Collaboration and communication are embedded in numerous care concepts, such as the chronic care model [3, 10] or the structuration model of interprofessional collaboration framework which offers a structured conceptual framework for understanding collaboration based on shared goals, internalisation, formalisation, and governance [11].

InCaD, characterised as structured, patient-centered communicative interactions involving at least two distinct health professions [9], are a possible tool for incorporating interprofessional knowledge and perspectives and jointly managing complex care situations [12]. InCaD enable those involved to pool their specific knowledge from different professions to make informed decisions and reflect on ethical issues [13].

### Various forms of InCaD

In our published systematic review [9] various forms of InCaD, such as interprofessional bedside rounds (I(B)Rs), interdisciplinary rounds (IDRs), structured interdisciplinary bedside rounds (SI(B)Rs), moral case deliberations (MCDs) and multidisciplinary team meetings (MDTMs), have been described [14–18]. But an established standard for their implementation and design is still lacking. The research literature shows a variety of models and approaches, but theory-based approaches that enable targeted adaptation and sustainable implementation are lacking [12, 13]. Hospital decision-makers tend to establish innovative formats of interprofessional collaboration in the short term without locating them theoretically or creating detailed method descriptions [19]. As a result, InCaD is being implemented in practice, but the realisation of InCaD varies greatly [20–22]. For example, I(B)Rs and MDTMs promote the involvement of patients and their families, promoting care coordination and positive treatment outcomes [22–24]. Structural and substantive differences between InCaD models often make it difficult for interprofessional teams to rely on a standardised approach and recognise their role in it. In nursing, InCaD are often not implemented as a tool of nurses’ own professional practice, with nurses often being only one participant among others in an InCaD initiated by other professional groups [16, 25, 26].

However, challenges arise not only in implementation but also in the organisational environment. A lack of resources and time for regular InCaD can affect their sustainability and effectiveness [27]. Although clinical decision-makers and nursing managers are increasingly recognising the value of InCaD, there is often insufficient institutional support, for example, in the form of appropriate structuring and prioritisation [28]. Cultural differences and hierarchical structures within healthcare teams make successful implementation more difficult [20, 29]. All these factors may impact communication and collaboration between different professional groups and lead to varying acceptance and efficiency of InCaD [30].

### Programme theory development

Although knowledge about different forms of InCaD has accumulated and is growing, uncertainties and inaccuracies in the design of InCaD as well as in their implementation have contributed to variances in the effectiveness of InCaD [13]For these reasons, the development of a theory-driven programme theory for InCaD is needed. Programme theory can help clinical decision makers understand the mechanisms and conditions under which InCaD works effectively and how it can be optimally integrated into everyday clinical practice [31]. A theory-based approach offers the opportunity to identify conditions for InCaD and thus to create a framework for the successful implementation and evaluation of InCaD. The advantage of programme theory for InCaD is its potential flexibility and adaptability [31]. InCaD is a key component for improving patient care because it promotes communication and collaboration between different professional groups, increases the quality of clinical decisions, and can structure and optimize the care of complex patient situations. Since requirements and structures in hospitals vary, a theory-driven approach enables the adaptation of case discussion formats to specific needs and circumstances of individual teams and patient groups [12]. A framework can increase the acceptance of InCaD within teams and promote interprofessional communication and collaboration. A common understanding and structured methodology can contribute to improving the quality of care and increasing patient safety [32, 33].

To close the gap of a missing theoretical anchoring of InCaD, we aim to develop a concept for the implementation and evaluation of an InCaD in a hospital at the programme theory level. Following a prospective realist methodology approach [34], we therefore ask an overarching research questions:

*“What works in InCaD in a hospital setting for whom in what circumstances and in what way*,* and how?”*

Our results can contribute to anchoring InCaD as an effective tool in clinical practice to achieve a higher quality of care and increased satisfaction for all those involved in the long term [14, 29, 35].

## Methods

**Fig. 1:**
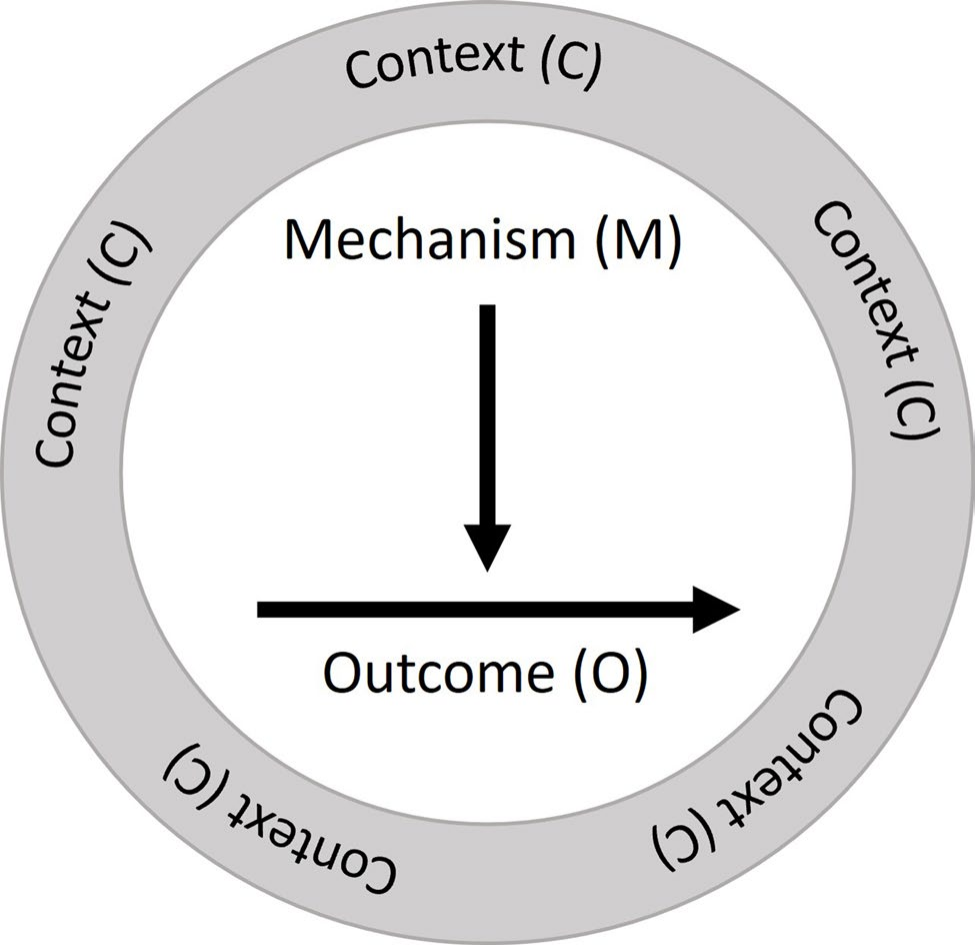
Classic realist context-mechanism-outcome-configuration [34]

To determine our aim and the development steps of InCaD, we referred to the methodological approach of the Medical Research Council (MRC) Framework [36, 37]. The MRC framework is an international gold standard for the development and evaluation of complex interventions in healthcare and nursing [31]. InCaD represents a complex intervention, as it involves multiple interacting components that function across various levels within healthcare systems. These components include the integration of interprofessional collaboration; adaptation to the dynamic needs of patients, families, friends and caregivers; and alignment with institutional priorities and resources. The complexity arises from the necessity of coordinating various stakeholders, addressing ethical and medical dilemmas, and maintaining structured communication amidst diverse professional cultures and hierarchies [38]. The design process of complex interventions according to the MRC framework acknowledges and considers the interplay of different related and interacting components [39, 40]. In addition, the application of programme theory to highlight how and under what conditions change is achieved by an intervention is recommended. Such theory explains the mechanisms underlying the programme, the characteristics of the context that should influence these mechanisms and how these mechanisms can influence the context [41]. To achieve this, we have chosen a prospective approach using the realist methodology and followed in this process the RAMESES II reporting standards for realist evaluations [42] (see Additional file 1). The realist methodology provides a framework for analysing complex interventions by examining how and why an intervention leads to the observed outcomes in specific contexts [43]. The realist methodology is based on realist philosophy, which assumes that interventions do not act in isolation but rather develop their effects through the behavior and decisions of the actors involved [44]. In contrast to traditional evaluation approaches, which focus primarily on the outcomes of an intervention, the realist methodology aims to identify the underlying mechanisms and to understand how these mechanisms are influenced by context [34, 45]. Although this methodology is generally used to evaluate existing programmes, a prospective approach allows programme theory to be aligned with a prior theoretical understanding of the mechanisms and chains of effects. Realist methodology research is guided by the following question: “What works for whom in what circumstances and in what way, and how?” [34, 46] The underlying cause‒effect relationships are formulated as context **(C)** mechanism **(M)** outcome **(O)** configurations (CMOs) (see Table 1 for definitions of key realist terms). In this context, the context is meant as a larger field, and if the conditions are right within that context, it triggers mechanisms that produce outcomes. These mechanisms are thought of as being triggered under the right contexts (see Fig. 1).

**Table 1:** Definitions of key realist methodology terms

Key term	Short description
*Programme*	A programme is typically multifaceted intervention or strategy aimed at driving change. The implementation involves diverse stakeholders, whose participation and interpretation of the provided resources play a critical role in shaping the programme’s outcomes [34].
*Programme theory*	A realist programme theory is a hypothetical framework, often articulated as an “if…then” statement, that explains how a programme or its components produce specific outcomes by identifying the mechanisms of change. These mechanisms are not the programme itself but the responses and reasoning of stakeholders or participants triggered by the programme under particular circumstances [47].
*Context*	Context refers to the conditions and settings in which a programme or intervention is implemented, including the characteristics of participants, stakeholder relationships, organizational environments, and cultural norms. It encompasses both the immediate institutional arrangements and broader societal, economic, or cultural factors influencing the programme’s implementation and outcomes [48].
*Mechanism*	Mechanisms are the processes or interactions triggered by an intervention’s resources, which lead to changes or outcomes in specific contexts. They involve participants’ cognitive, emotional, or motivational responses to these resources, are influenced by contextual factors, and are distinct from strategies, focusing instead on the generative processes that drive outcomes [48].
*Outcome*	Refer to the changes or effects that emerge from the interaction between contexts and mechanisms within a programme or intervention [48].
*Context-Mechanism-Outcome Configuration*	Is a central heuristic in realist evaluation, used to explain what works, for whom, under which circumstances, and how. It involves analysing the interaction of contexts, mechanisms, and outcomes, which can be linked in chains or embedded configurations to refine or generate programme theories [47].

### Development of programme theory

**Fig. 2:**
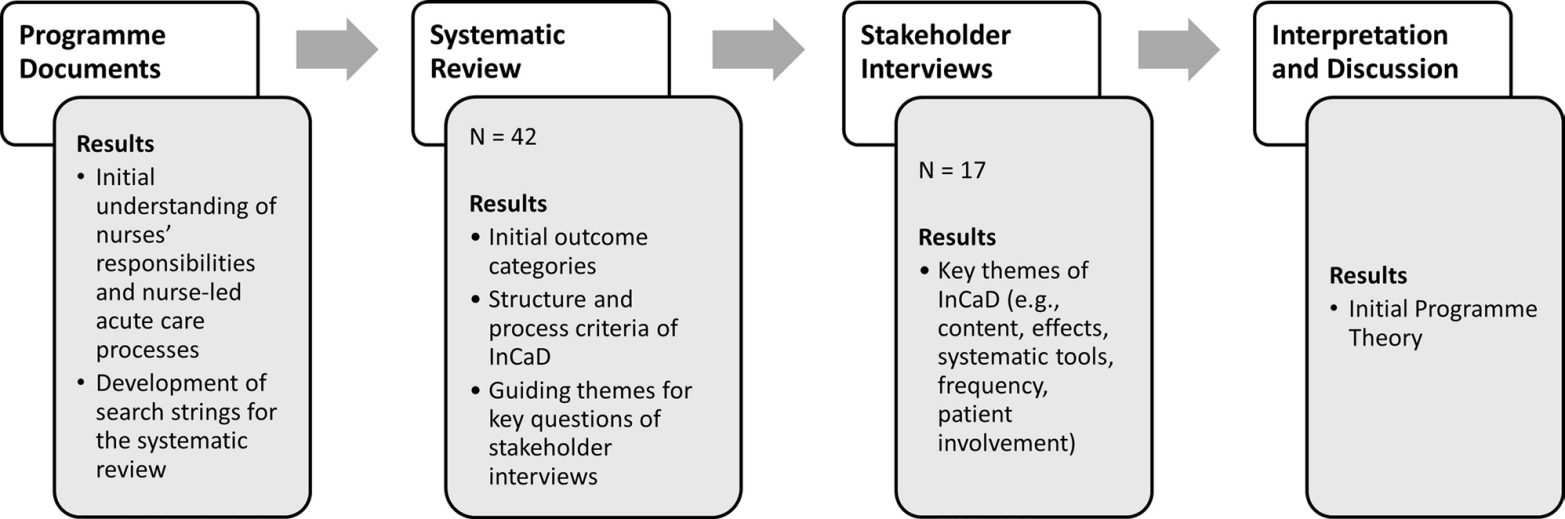
Process of the initial programme theory development

The development of our initial programme theory of the InCaD is based on three different data sources to include as many different perspectives as possible in the process. These data included findings from **(1)** an analysis of internal programme documents, (2) a systematic review [9] and **(3)** focus group interviews with hospital stakeholders and served to develop an initial understanding (I-III) of the possible intended effects and modes of action of the InCaD in an iterative process (see Fig. 2). A formal prioritisation or consensus technique (e.g., Delphi or ranking) was not applied. Instead, patterns were identified through thematic groupings and refined through discussion within the research team to ensure conceptual clarity and alignment with realist methodology [34].

1. Analysis of Internal Programme Documents.
  To gain insight into the theoretical foundations of the programme on the basis of the development process, the formulated objectives and the underlying principles, we analysed internal programme documents, including project planning documents, to gain an initial understanding of the possible responsibilities of nurses and nurse-led processes in acute care. The insights gained from these documents also served as a guide for the search strings in the systematic review and for the topics to be explored in depth in the interviews with the stakeholders.
2. Systematic Review.
  We then conducted a systematic review using the results of the document analysis as the basis for the research process. The systematic review, including all methodological appendices, has been published elsewhere [9], and summarises as follows. Systematic literature searches were conducted in January and December 2024 in PubMed, CINAHL, the Cochrane Library and PsycINFO and followed the PRSIMA 2020 guidelines [49] throughout the process. Studies that examine multiprofessional case discussions in inpatient hospital settings and in which nurses are involved as moderators or participants were included. Study selection and data extraction were organised via EndNote and Rayyan and carried out independently by two authors (JP & KS), achieving a kappa agreement of 0.89 for screening reliability. The quality assessment of the studies was performed at the level of evidence but without a risk assessment for bias. The screening of 4541 records led to the inclusion of 42 studies (PRISMA-Flowchart as Additional file 2). From the results, the first expected outcome categories, structure and process criteria of the InCaD and the key questions for the subsequent stakeholder focus group interviews were derived [9].
3. Stakeholder focus group interviews.
  As a third step, we conducted directive focus group interviews (*N* = 17) with different professional groups as hospital stakeholders (four physicians, five nurses, four nurse managers, and four therapists) (the sociodemographic characteristics of the participants are presented in Additional file 3). Participants received comprehensive written and verbal information before voluntarily participating in the focus groups. All participants provided written informed consent. Conduction of the focus groups was approved by the ethics committee of the German Society of Nursing Science (DGP eV.) on the 14th of April 2024 (application no. 24 − 007) as part of a larger study on the implementation of a nurse-led unit in a German maximum provider hospital.
  We aimed to capture different perspectives and gain deeper insights into the content, location, frequency, resources, duration, timing, participants and expected effects of the InCaD through group dynamics [50]. The interviews took place in March 2024 and lasted between 90 and 110 min. All the authors were involved in data collection. Using a semistructured guide specifically developed for this occasion (see Table 2 and Additional file 4). Interview questions were derived from the results from step 1 and 2 of the programme theory development process (see Fig. 2). We enabled a targeted but flexible discussion of the research topics [51]. To stimulate discussions, we used written and verbal stimuli and visualised participants’ key statements on presentation cards. After each topic, the participants had the opportunity to ask questions and review the results. In addition to audio recordings, we created field notes and conversation transcripts. After the audio data were transcribed verbatim, we conducted the analysis with MAXQDA (2024) software, which supports data analysis with diverse coding options, on the basis of Mayring’s qualitative content analysis [51]. We systematically coded the transcripts and identified key themes. The main categories were developed deductively via the interview guide while remaining open to the inductive development of new categories from the text data. We identified six key themes, such as the content of InCaD, effects, systematic tools, and the frequency or involvement of patients in InCaD.

### Data synthesis

We synthesised the data via realist logic by combining contexts **(C)**, mechanisms **(M)**, and outcomes **(O)** into CMO-configurations. Therefore, we formulate direct mechanisms of action, which refer to the direct cause‒effect relationship between a context and a result through the activation of a mechanism, and indirect mechanisms of action, which refer to mechanisms that do not act directly but lead to a result via intermediate steps [52]. To follow a logical process in formulating the CMOs, we followed Chen’s theory of action (ToA) [53] for data analysis in the initial category formation. According to ToA, a programme is only set in motion by an action model and will not work if it is based on invalid assumptions, is poorly constructed or unrealistic [53]. In a process of critical reflection and discussion, we created a logical model and developed an initial programme theory on the basis of the CMO heuristic. We used key questions for CMO-generation [44, 54] as a guide. On the basis of the identified CMOs, we developed in repeated rounds of discussions within the research team an initial programme theory to gain a preliminary understanding that clarifies how and why InCaD can exert its effects. To enhance the understanding of CMOs, which are often presented as if-then statements, we structured them as CMOs, describing the relationships in the form of “if – then – because” [55].

**Table 2:** Key questions stakeholder Interviews - Part of interprofessional case discussions

**Topic Introduction Interprofessional case discussions**	Many units worldwide have introduced special interprofessional case discussions to ensure good patient care. So far, little is known about what content is particularly important for such interprofessional case discussions, who should participate and how the case discussions affect the care process.
**Key Questions**	1. *If you look at these characteristics of case discussions*,* which characteristics should such a case discussion [on the nurse-led unit at this hospital] also contain and why?“* 2. *“In your opinion*,* how could such an interprofessional case discussion take place […]? When and how often does it take place?* 3. *Which people or professional groups should take part? What should be the objective of the interprofessional case discussion […]?* 4. *What content should be discussed in such an interprofessional case discussion? For which patients should the case discussions take place?* 5. *What impact do you think such case discussions […] could have for patients or staff? How do you think we would recognise that the case discussion […] is a good thing?*

## Results

The initial programme theory shows possible expected results of InCaD and explains the mechanisms and relationships that can be expected in the implementation of InCaD in clinical practice. Programme theory consists of formulated CMOs. For initial programme theory, we identified **(1)** what works for patients, families, friends and their caregivers, under what circumstances, in what ways, and how and **(2)** what works for the interprofessional team, under what circumstances, in what ways, and how, as the primary level of impact of an InCaD.

**Fig. 3:**
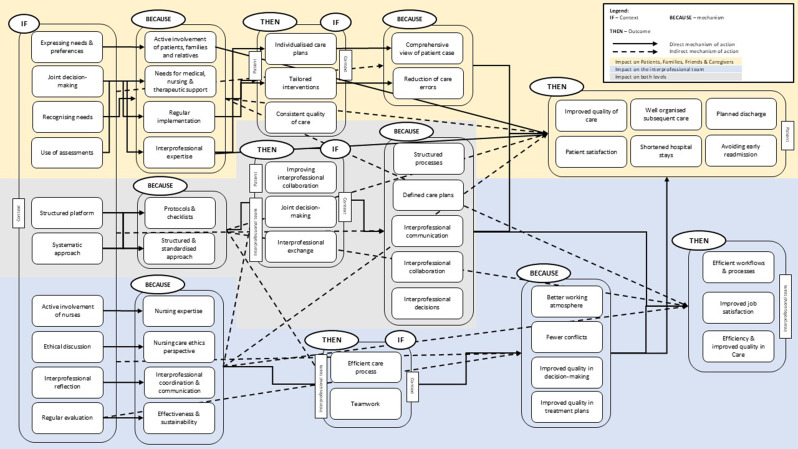
Context-Mechanism-Outcome Configuration for InCaD in inpatient care

For these two groups, we identified different direct and indirect mechanisms **(M)** of actions that can lead to positive outcomes **(O)** when viewed from different contexts **(C)** (see Fig. 3, two separate figures with separate direct and indirect mechanisms are in the Additional file 5). Additionally Table 3 provides an overview of the most prominent CMO-configurations identified. This shows how recurring contextual factors and mechanisms contribute to observed outcomes and highlights the dynamic interplay of collaboration, communication and role clarity within InCaD structures.

In total, we identified eight direct mechanisms of action and three indirect mechanisms of action for patients, families, friends and their caregivers. For the interprofessional team, we identified six direct mechanisms of action and five indirect mechanisms of action.

**Table 3:** Examples of CMO-configuration for InCaD

**Guiding question: “What works for patients, families, friends and their caregivers, under what circumstances, in what ways, and how?”)**
*Direct mechanism of action*
(1) **IF (C)** a joint decision-making process is applied, **THEN (O)** individualised care plans tailored to the health and psychosocial requirements of patients are developed, **BECAUSE (M)** patients’ different needs for medical, nursing and therapeutic support are considered.
(2) **IF (C)** a systematic approach and structured platform for InCaD are in place, **THEN (O)** quality of care, interprofessional exchange, and joint decision-making improve, **BECAUSE (M)** structured protocols and checklists are used.
(3) **IF (C)** patients, families, friends and their caregivers can express their own needs and preferences, **THEN (O)** their satisfaction in the treatment process is positively influenced, **BECAUSE (M)** they are actively involved in care.
**Guiding question: “What works for the interprofessional team**,** under what circumstances**,** in what ways**,** and how?”**
*Direct mechanism of action*
(1) **IF (C)** nurses are actively involved in InCaD, **THEN (O)** the efficiency of the care process and teamwork improves, **BECAUSE (M)** their professional perspective and leadership are integrated.
(2) **IF (C)** interprofessional reflection takes place, **THEN (O)** an efficient care process is achieved, **BECAUSE (M)** interprofessional coordination and communication are enabled.
(3) **IF (C)** efficient processes in care are established, **THEN (O)** workflow and working process becomes more efficient and job satisfaction increases, **BECAUSE (M)** a better working atmosphere and fewer conflicts result.

**(1) “What works for patients**,** families**,** friends and their caregivers**,** under what circumstances**,** in what ways**,** and how?”**

### Direct mechanism of action

InCaD **(C)** has numerous advantages for patients, families, friends and their caregivers. The regular implementation **(M)** of InCaD ensures a consistent quality of care **(O)**. This regularity creates a platform for individualised care plans and defined care requirements **(O)**, ensuring that patients’ needs are considered **(C)**. The quality of care improves **(O)** because professionals from different disciplines share their expertise **(M)** at regular intervals, enabling them to make joint decisions **(O)**.

A key mechanism of InCaD is the consideration of patients’ different needs for medical, nursing and therapeutic support **(M)**. Through a joint decision-making process **(C)**, the creation of individualised care plans that are tailored to the health and psychosocial requirements of patients **(O)** is enabled. In addition, the involvement of interprofessional expertise **(M)** helps to gain a comprehensive view of patient cases and to improve the quality of care **(O)**. This individualised approach ensures that patients and their families and friends receive individualised care **(C)**, because of a comprehensive view of the patient case **(M)**, which ultimately supports the patients’ recovery and satisfaction **(O)**.

Another mechanism of InCaD is the active involvement of patients, families, friends and their caregivers **(M)** in the decision-making process related to their care. This creates the opportunity to express their own needs and preferences **(C)**, which can positively influence their satisfaction **(O)** in the treatment process.

If it is not possible to actively involve the patients themselves, it is important to recognise their needs **(C)**. When assessments are used to determine specific care needs **(C)**, patients who are not able to express their preferences and actively participate in care-making-decisions receive care that meets their individual needs **(O)** because their needs can be actively involved in the care process. Through the use of these assessments, a structured way **(M)** of recording care needs and developing tailored interventions **(O)** can be offered.

Another important element of InCaD is the use of structured protocols and checklists **(M)**. These tools ensure a systematic approach and structured platform for InCaD **(C)**, which can lead to improved quality of care, interprofessional exchange and a joint decision-making process **(O)**. These aspects **(C)** can also lead to planned discharges or shortened hospital stays **(O)** through interprofessional communication and collaboration **(M)**. It also enables clearly documented and traceable decision-making **(O)**, which can increase patient safety and improve the overall quality of care **(O)**.

The structured and systematic approach **(C)** of InCaD also helps to ensure that discharge planning and subsequent care are well organised and that potential gaps in care after a hospital stay are minimised **(O)**. Additionally, the risk of an early readmission can be reduced, which in turn increases the satisfaction of patients, families, friends and their caregivers **(O)**.

### Indirect mechanisms of action

As part of the initial programme theory, we were able to identify direct mechanisms of action. However, as shown in Fig. 3, these are not the only factors that characterise the process and outcomes of InCaD. Various CMO- configurations have also been identified, which can currently be assumed to represent an indirect mechanism of action and do not operate in the direct cause‒effect relationships described.

For the outcome level of patients, families, friends and their caregivers, it can be assumed that if their expression and ability to recognise their needs, joint decision-making, and the use of assessments **(C)** are defined as the context for the InCaD, an influence can be exerted on possible outcomes such as improved quality of care, planned discharge or patient satisfaction **(O)**. In this context, comprehensive care of the patient and a reduction in care errors **(M)** act as mechanisms.

However, it is not only possible to identify indirect mechanisms of action that affect only patients, families, friends and their caregivers but also influence outcomes such as efficient workflow or efficiency and quality in care **(O)** at the level of the interprofessional team. This cause‒effect relationship can be recognised when patients, families, friends and their caregivers are actively involved; their needs for medical, nursing and therapeutic support are included in the InCaD; and interprofessional expertise **(M)** is also included in the InCaD. These mechanisms must also be considered in terms of their outcome effects at the patient, family, friend and caregiver levels.

(2) “What works for the interprofessional team, under what circumstances, in what ways, and how?”

### Direct mechanism of action

For the interprofessional team, InCaD also represents a platform that promotes cooperation and communication between the professionals involved **(O)**. A structured and systematic approach **(M)** is also a central mechanism of InCaD that supports interprofessional exchange and joint decision-making within the team **(O)**. This interprofessional approach involves different disciplines, where a dynamic of learning and exchange is created that can strengthen teamwork and aim for an efficient process in care **(O)**. Furthermore, if nurses are actively involved in InCaD and ethical discussions are held **(C)**, this can also have an impact on the efficiency of the care process and teamwork **(O)**, as nursing expertise and nurses’ ethical perspective **(M)** play important roles in the care of patients, and these are considered.

Additionally, a systematic approach **(C)**, which is supported by structured procedures, checklists and protocols **(M)**, can also improve the efficiency of collaboration **(O)**. Interprofessional exchange **(C)** makes it easier for interprofessional team members to prioritise the most important issues and to make joint decisions **(O)**. Efficient processes in care **(M)** not only promote an efficient workflow **(O)** but also increase satisfaction within the team **(O)**, as a better working atmosphere and fewer conflicts **(M)** are gained. The satisfaction of team members is reflected in improved team dynamics and greater motivation to contribute to joint care provision **(O)**.

When everyone is involved in interprofessional reflection **(C)**, the team can make joint decisions **(O)** on the basis of a shared understanding of the patient’s case **(M)**. This not only improves the quality of decision-making but also efficiency, as unnecessary repetition and misunderstandings in the treatment process can be avoided **(O)**. This standardised information base **(C)** promotes a sense of togetherness and can reduce feelings of frustration **(M)** that may arise when information is unclear or unavailable.

In addition, the active involvement of nurses **(C)** in ethical discussions and reflections on complex cases improved the working atmosphere **(O)**. Ethical discussions enable team members to incorporate different perspectives and values into the decision-making process **(M)**, which creates the basis for collaborative, responsible decision-making **(C)**. Regular interprofessional exchange **(M)** makes it possible to reduce conflicts within the team **(O)**, as teamwork **(C)** is promoted. When conflicts are minimised and teams stand by their decisions **(M)**, the working atmosphere and general team cohesion improve **(O)**.

### Indirect mechanisms of action

As with the impact of InCaD on patients, families, friends and their caregivers, we were also able to identify direct mechanisms of action for the impact on the interprofessional team.

If nurses are actively involved in InCaD **(C)**, for example, in leadership roles, this can have a positive impact not only on their professional satisfaction but also on their workflow efficiency and quality of care **(O)**. However, the inclusion of ethical discussions and interprofessional reflection **(C)** may also have an impact on efficiency and quality in care as well as job satisfaction **(O)** because of the resulting better working atmosphere, fewer conflicts or quality in decision-making **(M)**.

With respect to these outcomes, regular evaluations **(C)** represent a further indirect mechanism of action, such as improving the working atmosphere, fewer conflicts and changes in the quality of decision-making and treatment plans **(M)**.

InCaD, nurses are offered the opportunity to contribute their nursing expertise and their ethical perspective **(M)** to increase the efficiency of nursing processes **(C)**. The active involvement of nurses in InCaD **(M)** not only increases nursing expertise on the team but also promotes job satisfaction **(O)**. Particularly in complex decision-making situations in which ethical discussions play a central role **(C)**, the involvement of nurses **(M)** can contribute to improving the working flow and efficiency and quality of care **(O)**.

Furthermore, the CMO-configurations cannot be considered only in terms of their impact on the interprofessional team. It can be assumed that contexts such as ethical discussions, interprofessional reflection **(C)** and mechanisms such as interprofessional coordination and communication or the involvement of the nursing care ethics perspective **(M)** have an indirect influence on the outcomes of patients, families and caregivers **(O)**.

### Summary

InCaD can offer benefits for patients, families, friends and their caregivers as well as for the interprofessional team. The regular implementation and systematic organisation of InCaD contribute to a high quality of care. For patients, this can result in tailored care plans that consider their medical, nursing and therapeutic needs and thus ensure holistic care. The active involvement of patients, patients, families, friends and their caregivers makes it possible to integrate personal needs into the decision-making process, which can significantly increase patient satisfaction. Even in situations where patients cannot actively participate in InCaD, the use of various assessment instruments ensures that care remains needs-oriented and individual. These measures improve patient safety and promote clear and consistent care planning, which can streamline hospital discharge and reduce the risk of readmission.

For interprofessional teams, InCaD create an opportunity for exchange and collaboration between different healthcare professionals, promoting efficiency and satisfaction within the team. The utilisation of structured protocols and checklists promotes uniformity and transparency, which can strengthen the trust and motivation of team members. Nurses have the opportunity to contribute their professional expertise and ethical perspective to the decision-making process, which can increase their professional satisfaction and acknowledge their expertise within the team. Regular interprofessional communication grants everyone involved access to information, thereby minimising misunderstandings and conflicts. This promotes a harmonious working atmosphere and can improve team cohesion, which ultimately further optimises patient care.

Overall, our results reveal which contextual factors and mechanisms influence which outcomes and provide an explanation of how InCaD can have an effect on patients, families, friends and their caregivers and the interprofessional team. It is a method for improving the quality of care, patient satisfaction and professional fulfillment of the interprofessional team through the systematic integration of different types of expertise and the involvement of patients, families, friends and their caregivers.

## Discussion

This study advances existing knowledge on InCaD and its potential impact by employing a realist approach [34] to analyse the factors influencing its implementation and effectiveness in hospital settings. These findings emphasise that the mechanisms driving the success of InCaD are highly context dependent. These contexts can vary not only between different healthcare institutions but also within interprofessional teams. This insight underscores the importance of tailored strategies that align with specific institutional and professional conditions.

The presented CMO-configurations synthesise the core mechanisms by which InCaD influences both patient, families, friends and their caregivers outcomes and interprofessional team functioning. For patients, key contextual conditions such as structured decision-making or participation opportunities lead to improved satisfaction and care quality, especially when underpinned by a systematic approach. For interprofessional teams, mechanisms like nurse leadership or interprofessional reflection foster process efficiency and team cohesion. These configurations serve to clarify the possible pathways of InCaD’s effects.

A central aspect of this study is the identification of direct and indirect mechanisms of action operating across different levels [52]. Direct mechanisms, such as the structured use of checklists; the promotion of interprofessional reflection; and the active involvement of patients, families, friends and their caregivers and nurses in decision-making processes, have immediate impacts on the quality of care. These mechanisms facilitate the development of individualised care plans, enhance teamwork, and improve workflows and efficiency in care. Direct mechanisms such as the use of structured and transparent documentation and shared decision-making are also recognized as essential drivers of quality improvement interventions, as they ensure a consistent and accessible information flow across teams [56]. Indirect mechanisms, on the other hand, include fostering a regular evaluation and integrating ethical perspectives into decision-making. These mechanisms are similar to those found in broader quality improvement initiatives, where fostering a culture of continuous reflection and establishing feedback loops are key factors in achieving sustainable changes in healthcare settings [39]. These mechanisms influence team dynamics over the long term and support a better working atmosphere or quality in decision-making, contributing to the sustainable integration of InCaD as a core element of care. Such processes play a critical role in shaping the dynamics and effectiveness of collaboration within interprofessional teams. Furthermore, patient-related outcomes, such as satisfaction and continuity of care, are common endpoints used to measure quality improvements in healthcare interventions, which further emphasizes the broader applicability of InCaD beyond their immediate context [57].

The interactions between these identified mechanisms are complex and context dependent. While direct mechanisms have an immediate influence on specific processes, indirect mechanisms shape values, norms, and system-wide dynamics over the long term. This finding highlights crucial insight into other quality improvement interventions, where local, smaller changes often drive larger system-wide transformations [39]. Nonetheless, the findings highlight that system-wide improvements often emerge from the interplay of smaller, localised processes. This dynamic not only enhances the quality of patient care but also fosters a culture of continuous reflection and learning within interprofessional teams. The establishment of a reflective learning culture is a critical component of sustained healthcare improvement, as it fosters ongoing learning, shared understanding, and continuous adaptation to evolving clinical demand [57]. Such a culture is potentially important for strengthening interprofessional collaboration and ensuring the long-term satisfaction of the involved professionals. Notably, staff-related outcomes such as job satisfaction, teamwork coherence, and a sense of professional identity are increasingly recognized as essential for improving workforce retention and mitigating the impact of staffing shortages.

Our work provides a critical starting point for testing and further developing theoretical assumptions about how InCaD functions. Notably, the methodological approach, which combines diverse data sources, offers a nuanced perspective on the relationships between contexts, mechanisms, and outcomes. Future studies should aim to validate and refine the proposed CMO-configurations in clinical and international settings to develop a more comprehensive understanding of the conditions and processes under which InCaD achieves lasting improvements. The ultimate goal is to establish a middle-range theory that facilitates broader applicability of these findings. This approach aligns with existing research on quality improvement interventions, where context-sensitive implementation strategies have been shown to increase the relevance and transferability of intervention models to different healthcare settings [36].

### What’s in the evidence?

Even if the evidence provides a guiding basis for our results of the CMO- configuration, these results need to be discussed with each other at this stage. We were able to demonstrate the active involvement of patients, families and their friends in InCaD as a mechanism that has a positive effect on the outcomes of those affected. The empirical analysis highlights that formats such as I(B)Rs and MDTMs, which promote the involvement of patients and their friends, can support better coordination and positive treatment outcomes [20, 24]. Furthermore, the evidence shows that only InCaD that takes place over a longer period of time and in a larger group of participants are more likely to involve friends [15, 58]. However, our results highlight the need for structured integration of friends and families in the design of InCaD, particularly in cases where the short-term nature of the InCaD makes deeper involvement difficult. Here, the use of assessments and protocols can be used to systematically involve friends to ensure that their perspectives and needs are considered in care decisions.

The positive impact that InCaD could have on patient satisfaction and the interprofessional team as outcomes according to our initial programme theory is consistent with the evidence, which also emphasises the role of interprofessional collaboration in improving the quality of care [59–61]. While previous studies have mostly examined the functional effects of interprofessional collaboration on patient outcomes and have often focused on optimising communication and increasing efficiency [14, 28, 35], by applying the realist methodology in this study, we achieved a differentiated perspective on the mechanisms and contexts that could lead to the expected effects in different settings.

### From participant to leader: active involvement of nurses in InCaD

Another important factor is the opportunity for nurses to take over the management of InCaD, which can increase their professional autonomy and self-confidence. Taking on this responsible role promotes confidence in one’s own abilities and acknowledges their expertise within the team. The nurse-led organisation and management of the InCaD gives nurses a stringer sense of making a responsible contribution to the care process that they can guide and positively influence, which can have a positive impact on their job satisfaction and the quality of their work.

With respect to the involvement of nurses, the research literature shows that they rarely take primary responsibility or facilitation roles in InCaD. Instead, physicians often dominate the facilitation, possibly reflecting reservations about expanded nurse competencies [26, 28]. However, our findings suggest that the active involvement of nurses in InCaD could not only promote their clinical competence through exchange with other professional groups but also contribute to their professional development. This highlights the potential of InCaD as a tool for professional and clinical development, as nurses expand their skills and knowledge through regular participation. Another aspect of our findings that is rarely covered in the literature is the use of nurse-led InCaD [17, 59, 61, 62]. We hypothesise that the integration of nurse-led InCaD can have a positive effect on job satisfaction and workload by promoting better care and communication.

This nurse-led care approach has already been implemented in practice at a relatively high level. Within the framework of nurse-led care, nursing staff are primarily responsible for patient care, and medical staff are involved only on a consultative basis. Nurse-led units (NLUs) are one type of implementation that already exists. NLUs are considered wards where the admission decision for patients is made by nurses and not by medical staff and where nursing is the main therapy. In addition, professional nurses are considered leaders of the clinical team and are authorised not only to admit patients but also to make discharge decisions [63–65]. In this context, nurse-led InCaD represents a possible approach to expand this type of care even more specifically through nurses.

### Methodological reflection

This initial programme theory, which uses the realist methodology to develop hypotheses about the potential effects and modes of action of the InCaD, proves to be a valuable basis for systematically analysing the relationships between the specific contexts, mechanisms and expected results of the InCaD. This methodology enables us to capture a differentiated record of the complex interventions, potential results and underlying mechanisms and their context dependency, whereby the effect can not only be described but also interpreted in a structured manner on the basis of the respective framework conditions, and a hypothetical approach to conditions under which InCaD might be particularly effective in practice is possible [34].

However, the interpretation of the CMO-configuration is challenging, especially in highly heterogeneous contexts, as the diverse framework conditions and team structures make the generalisation and standardisation of hypotheses difficult [37]. Nevertheless, it is important to follow this approach. In particular, considering the existing evidence, in which various positive effects of InCaD can be identified but no generally valid approach is followed, this systematisation within the framework of programme theory is essential to be able to make generally valid statements in the future. For this reason, we also followed Chen’s ToA [53] to evaluate why InCaD might work in practice and why it might not.

### Limitations

Although the results provide valuable insights, some limitations need to be considered. First, the results are based on assumptions derived from the document analysis, systematic review and stakeholder interviews. These assumptions represent hypotheses that need to be tested in further studies. However, this was our aim, as we were pursuing a prospective approach with initial programme theory and not evaluating an existing programme. Second, the context dependency of the InCaD presents a challenge for generalisability. In different clinical settings, different settings, team compositions and cultural factors can influence the effectiveness of InCaD. Future studies should aim to analyse specific contextual factors systematically and assess their importance for CMO-configurations.

### Theoretical implications and evaluation of programme theory

The results and hypotheses of this study provide valuable guidance for further developing initial programme theory for implementing InCaD in clinical practice. The application of the realist methodology made it possible to formulate assumptions about the conditions and mechanisms under which InCaD is potentially effective. The identified CMO-configurations provide a theoretical basis for understanding how specific contexts and mechanisms might lead to the desired outcomes [43].

In the next step, the assumptions and results of this work will be used to conceptualise InCaD in a German hospital to evaluate programme theory via the realist evaluation approach. The targeted further development and validation of the theory can contribute to the development, design and implementation of InCaD in clinical practice and support long-term implementation in practice.

### Implications for practice

The CMO-configurations derived from the analysis suggest that regular integration of InCaD into clinical practice can be beneficial for continuous quality care and interprofessional collaboration. The assumption that regular implementation could serve as a central mechanism to ensure that all relevant professionals are always informed and on the same level of knowledge indicates the potential of InCaD to contribute to consistency and flexibility in care planning. Such regularity can promote efficiency and adaptability in dynamic clinical situations and increase trust and effectiveness in decision-making processes.

In addition, the CMO-configurations, exemplified in Table 3, offer opportunities to understand the mechanisms of action of InCaD in a differentiated manner. This creates an opportunity to define one’s own goals for implementation in practice, which are already explained with an effect-context relationship. This also allows decisions to be made for or against various aspects of the implementation.

### Implications for research & policy making

The CMO-configurations derived from the analysis provide the basis for future studies that could further investigate the assumptions about the effectiveness and sustainability of InCaD. Through an evaluation and revised programme theory, the generalisability of the hypotheses can be achieved, and at the same time, the importance of specific contextual factors for the effectiveness of InCaD can be illuminated. It becomes clear which adjustments are required in different clinical settings to achieve the best possible effectiveness.

For policymakers and hospital management, this underscores the necessity of establishing InCaD as a strategic tool to increase quality of care and staff satisfaction. Strengthening support for interprofessional approaches could not only improve patient safety and satisfaction but also, in the long term, increase the efficiency and attractiveness of healthcare facilities.

## Conclusion

This study provides a foundational framework for understanding and implementing InCaD in acute hospital settings. Using a realist methodology, the research identifies key CMO-configurations that optimize InCaD effectiveness.

Findings highlight that structured reflection, shared decision-making, and systematic protocols improve care quality and patient satisfaction. Additionally, fostering team trust and a culture of learning enhances team cohesion and job satisfaction. Nurse leadership and context-specific adaptations are crucial for sustainable implementation.

This study bridges theoretical frameworks and practical applications, offering actionable insights for healthcare leaders to improve patient safety, care quality, and staff well-being. By guiding systematic implementation, this work supports the integration of InCaD as a transformative tool in modern healthcare strategies.

## Supplementary Information

Below is the link to the electronic supplementary material.

Supplementary Material 1

Supplementary Material 2

Supplementary Material 3

Supplementary Material 4

Supplementary Material 5

Supplementary Material 6

## Data Availability

The dataset of the systematic review can be made available upon reasonable request from the authors. Data included in the stakeholder focus group interviews cannot be shared openly, to ensure participant privacy, but may be made available through the authors upon reasonable request and obtainment of consent from the participants. Data are stored in a controlled access data storage at the University of Applied Sciences Bremen.

## Abbreviations

CMO: Context-Mechanism-Outcome
DGP: German Society of Nursing Science
I(B)Rs): Interprofessional bedside rounds
IDRs: Interdisciplinary rounds
InCaD: Interprofessional case discussion
MCDs: Moral case deliberations
MDTMs: Multidisciplinary team meetings
MRC: Medical Research Council
SI(B)Rs: Structured interdisciplinary bedside rounds
ToA: Theory of Action
