# Supplementary material for: Initial programme theory developing for interprofessional case discussions (InCaD) in acute hospital care: a realist approach
Source: BMC Health Serv Res. 2025 Dec 11;26:21. doi: 10.1186/s12913-025-13865-5 (PMC12771898; doi:10.1186/s12913-025-13865-5)
Supplement: Supplementary file 1 — Supplementary Material 1 [file 12913_2025_13865_MOESM1_ESM.pdf]

*Supplementary Table 2: Sociodemographic characteristics of participants (N=17)*

| Characteristic | N |
|---|---|
| **Age-group** | |
| **< 25 years** | 1 |
| **25 to under 35 years** | 1 |
| **35 to under 45 years** | 3 |
| **45 to under 55 years** | 7 |
| **55 to under 65 years** | 4 |
| **≥ 65 years** | 1 |
| **Sex** | |
| **female** | 10 |
| **male** | 7 |
| **Duration of working in the healthcare sector** | |
| **Still in training** | - |
| **< 1 year** | - |
| **1 to 5 years** | 1 |
| **6 to 10 years** | 1 |
| **11 to 20 years** | 5 |
| **≥ 20 years** | 10 |
| **Highest job qualification** | 10 |
| **3 years of vocational training** | - |
| **B.A/B.Sc.** | 3 |
| **Diploma/M.A./M.Sc.** | 4 |
| **a state examination in medicine** | |
| **Work focus** | |
| **Direct nursing care** | 5 |
| **Nursing management** | 4 |
| **Medicine** | 4 |
| **Therapeutic professions** | 4 |
| **Duration of working in the study hospital** | |
| **< 1 year** | - |
| **1 to 5 years** | 2 |
| **6 to 10 years** | 1 |
| **11 to 20 years** | 6 |
| **≥ 20 years** | 8 |
