# Supplementary material for: Initial programme theory developing for interprofessional case discussions (InCaD) in acute hospital care: a realist approach
Source: BMC Health Serv Res. 2025 Dec 11;26:21. doi: 10.1186/s12913-025-13865-5 (PMC12771898; doi:10.1186/s12913-025-13865-5)
Supplement: Supplementary file 2 — Supplementary Material 2 [file 12913_2025_13865_MOESM2_ESM.pdf]

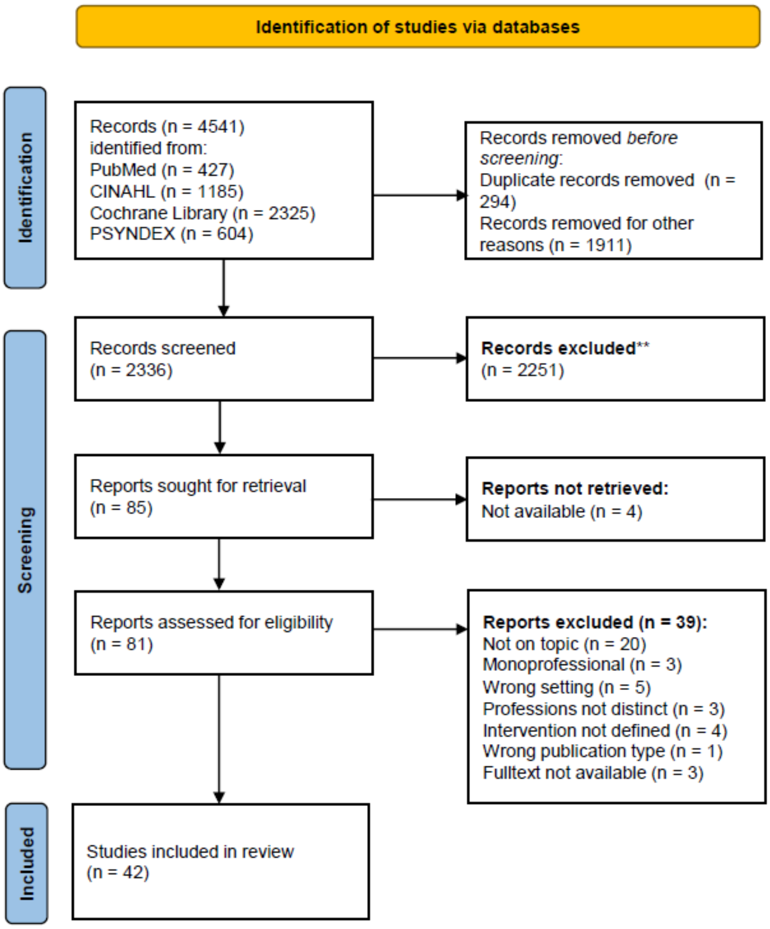

Supplementary Figure 1: PRISMA-Flowchart (Pöhner J, Regelmann EM, Seibert K, Stanze H. A systematic review on content, structure and process characteristics of interprofessional case discussions (InCaD) involving nurses in adult acute hospital care. J Interprof Care. 2025:1-17.)
