# Supplementary material for: Initial programme theory developing for interprofessional case discussions (InCaD) in acute hospital care: a realist approach
Source: BMC Health Serv Res. 2025 Dec 11;26:21. doi: 10.1186/s12913-025-13865-5 (PMC12771898; doi:10.1186/s12913-025-13865-5)
Supplement: Supplementary file 3 — Supplementary Material 3 [file 12913_2025_13865_MOESM3_ESM.pdf]

Procedure plan for stakeholder focus group interviews

Moderator Guidelines

Avoid making content-based statements

Engage the entire group during interventions

Address questions to the group rather than individuals

Minimise individual communication between participants and the moderator

<table>
<tr><th>Content</th><th>Discussion prompts, guiding questions, comments</th><th>Materials</th></tr>
<tr><td>Preparation</td><td>Display wall posters of key topics. Provide water, apple juice, glasses, snacks, etc.</td><td rowspan="2">Wall posters of topics, Flipcharts/Metaplan boards, Moderation cards, Markers, Tape/pins, PowerPoint slides, Consent forms, Information letters, Pens, Recording device</td></tr>
<tr><td>Welcome and Introductions
Presentation of study team and procedure/ moderation procedure, break regulations, handling of data protection and consent</td><td>!Start of audio recording after information on data protection and informed consent!</td></tr>
<tr><td>Presentation of the participants</td><td>Name, occupational group and specialisation, how long you have worked in the hospital, 3 short sentences on experiences with interprofessional cooperation in the hospital</td><td>Recording device</td></tr>
<tr><td>Presentation of NLU study and definition of NLU</td><td>Present study for a maximum of 7 minutes, follow-up questions, buffer time</td><td>PPT,
Recording device</td></tr>
</table>

**Starting the discussion**	*'We are now starting the discussion. We have three topics that we would like to discuss with you (please refer to the wall attachments). We have brought along some information on each topic, which we would like to present to you and ask you questions about. And we will start with the visionary topic of staff mix and nursing care on the NLU ward.'*	Wall posters of topics, Flip-charts/Metaplan boards, Moderation cards, Markers, Tape/pins
**Case discussions and interprofessional col-laboration***	**<u>Stimulus template</u>**<br>*'Many units worldwide have introduced special interprofessional case discussions to ensure good patient care. So far, little is known about what content is particularly important for such interprofes-sional case discussions, who should participate and how the case discussions affect the care pro-cess'. (READ list)*<br><br>**<u>Key Questions</u>**<br>1. *'If you look at the characteristics of case discussions, which characteristics should such a case discussion on the NLU at the KBR also contain and why?'*<br>2. *'In your opinion, how could such an interprofessional case discussion take place on the NLU at the KBR? When and how often does it take place?'*<br>3. *'Which people or professional groups should take part? What should be the objective of the interprofessional case discussion in the NLU at the KBR?'*<br>4. *'What content should be discussed in such an interprofessional case review? For which pa-tients should the case discussions take place?'*<br>5. *'What impact do you think such case discussions on the NLU at the KBR could have for the patients or for the staff? How do you think we would recognise that the case discussion on the NLU is a good thing?'*	<u>STIMULUS:</u><br>Contents and struc-tures of case dis-cussions in the lit-erature<br>(1x per participant, A4)
**Open question at the end of the discussion**	*'We now have about a quarter of an hour left. What points do you find particularly important with re-gard to today's discussion? What are the most important aspects (or tips) that you would like to give us for the NLU station?'*<br>**<u>If applicable:</u>** *'What else is particularly important to you about NLU in the KBR that we haven't talked about yet?'*	
**Farewell and further in-formation**	*‚Thank you very much for the interesting discussion. We will end the tape recording at this point.'*<br>What is the next step in the study? Where and when are the results available? When will we be in contact again?<br>Fill in short questionnaire	

**Post-processing**	Photo documentation Metaplan/Flipchart<br>Backup audio file<br>Short documentation Focus group<br>Documentation Pseudonyms	Camera<br>Laptop<br>Document: Short docu-mentation
*) Two upstream blocks of questions on participants' expectations and requirements for the organisation of care at the NLU are not the subject of the research reported here and are therefore not included here.		

Short Questionnaire

Please answer the following questions about yourself and your workplace so that we can make statements about the composition and representativeness of the group interviews. Your information will only be used anonymously.

Supplementary Table 4: Short Questionnaire

**1. How old are you?**	
☐ under 25 years	☐ 25 to under 35 years
☐ 35 to under 45 years	☐ 45 to under 55 years
☐ 55 to under 65 years	☐ 65 years and over
**2. How long have you been working in the healthcare sector (without training)?**	
☐ I am still in training	☐ 6 to 10 years
☐ less than 1 year	☐ 11 to 20 years
☐ 1 to 5 years	☐ more than 20 years
**3. What is the highest (professional) qualification you currently hold?**	
☐ 3-year vocational training without academic qualification	
☐ BA. / B.Sc.	
☐ Diploma/ MA. / M.Sc.	
☐ State Examination in Medicine	
☐ Doctoral degree	
☐ other: ______________________________	
**4. Which area of work best corresponds to your job?**	
☐ direct nursing care	
☐ Nursing management	
☐ Medicine	
☐ therapy professions (ergotherapy, physiotherapy, speech therapy and others)	
☐ another work focus: ______________________________	
**5. How long have you been working at the Bremerhaven Reinkenheide Clinic ?**	
☐ less than 1 year	☐ 11 to 20 years
☐ 1 to 5 years	☐ more than 20 years
☐ 6 to 10 years	
**6. Which department do you belong to?**	
______________________________	
**7. Which gender do you identify as?**	
☐ female ☐ male ☐ diverse	

Deutsche Version: Ablaufplan Fokusgruppendiskussion

Hinweise für die Moderation

Unterlassung inhaltlicher Stellungnahmen

Ansprechen der gesamten Gruppe bei Interventionen

Fragen nicht an Einzelne, sondern an das Kollektiv richten

Vermeidung der Individualkommunikation von einzelnen Teilnehmern mit dem Moderator

<table>
<tr><th>Inhalt</th><th>Diskussionsgenerierender Stimulus, Leitfragen, Kommentar</th><th>Material</th></tr>
<tr><td>Vorbereitung</td><td>Wandanhänge Themenfelder aufhängen, Wasser, Apfelsaft, Gläser, Snacks o.ä. breitstellen</td><td rowspan="2">Wandanhänge Themenfel-der,
Flipchart/ Metaplan,
Moderationskarten,
Eddings,
Klebeband/ Pins,
PPT,
Einwilligung Blanko,
Infoschreiben,
Kugelschreiber,
Aufnahmegerät</td></tr>
<tr><td>Begrüßung
Vorstellung Studienteam und Ablauf/ Moderations-ablauf, Pausenregelung, Umgang Datenschutz und Einwilligung</td><td>!Beginn der Tonaufzeichnung nach Hinweis zu Datenschutz und Einwilligung!</td></tr>
<tr><td>Vorstellung der Teil-nehmenden</td><td>Name, Berufsgruppe und Tätigkeitsschwerpunkt, seit wann Mitarbeiter im Krankenhaus, 3 kurze Sätze zu Erfahrungen mit interprofessioneller Zusammenarbeit im Krankenhaus</td><td>Aufnahmegerät</td></tr>
<tr><td>Vorstellung NLU-Studie und Definition NLU</td><td>Maximal 7 Minuten Studie vorstellen, Nachfragen, Pufferzeit</td><td>PPT,
Aufnahmegerät</td></tr>
</table>

**Einstieg in die Diskussion**	*„Wir starten jetzt in die gemeinsame Diskussion. Wir haben drei Themenfelder, die wir mit Ihnen diskutieren möchten (auf Wandanhänge verweisen). Wir haben zu jedem Thema ein paar Informationen mitgebracht, die wir Ihnen vorstellen und zu denen wir Fragen an Sie richten möchten. Und wir starten visionär mit dem Thema Personal-Mix und Pflegerische Versorgung auf der NLU-Station.“*	Wandanhänge Themenfelder, Moderationskarten, Edding, Klebeband
**Fallbesprechungen und interprofessionelle Zusammenarbeit***	**Vorlage Stimulus**<br>*„Viele Stationen auf der Welt haben besondere interprofessionelle Fallbesprechungen eingeführt, um eine gute Versorgung der Patient:innen zu sichern. Bislang ist aber wenig dazu bekannt, welche Inhalte für solche Fallbesprechungen besonders wichtig sind, wer sich daran beteiligen sollte und wie sich die Fallbesprechungen im Versorgungsprozess auswirken.*<br>*Hier sehen Sie eine Liste von möglichen Inhalten und Strukturen für Fallbesprechungen, die weltweit so beschrieben werden“. (Liste VORLESEN)*<br>**Leitfragen**<br>6. *„Wenn Sie sich die Merkmale von Fallbesprechungen anschauen, welche Merkmale sollte auch so eine Fallbesprechungen auf der NLU am KBR beinhalten und warum?“*<br>7. *„Wie könnte Ihrer Meinung nach so eine interprofessionelle Fallbesprechung auf der NLU am KBR ablaufen? Wann und wie oft findet sie statt?*<br>8. *Welche Personen oder Berufsgruppen sollten daran teilnehmen? Welche Zielsetzung sollte die interprofessionelle Fallbesprechung auf der NLU am KBR haben?“*<br>9. *Welche Inhalte sollten in einer solchen interprofessionellen Fallbesprechung besprochen werden? Für welche Patient:innen sollten die Fallbesprechungen stattfinden?*<br>10. *„Was glauben Sie, welche Auswirkungen können solche Fallbesprechungen auf der NLU am KBR für die Patient:innen oder für die Mitarbeitenden haben?*<br>*Woran würden wir Ihrer Meinung nach merken, dass die Fallbesprechung auf der NLU eine gute Sache ist?“*	STIMULUS:<br>In der Literatur Inhalte und Strukturen von Fallbesprechungen<br>(1x Pro Teilnehmer:in, A4)
**Offene Frage zum Abschluss der Diskussion**	*„Wir haben jetzt noch etwa eine viertel Stunde Zeit. Vor dem Hintergrund der heutigen Diskussion – welche Punkte finden Sie besonders wichtig? Was sind die wichtigsten Aspekte (oder Hinweise) die Sie uns für die NLU-Station mit auf den Weg geben wollen?“*<br>**Ggfs.:** *„Was ist Ihnen zu den Themen NLU am KBR noch besonders wichtig, worüber wir noch nicht gesprochen haben?“*	
**Verabschiedung und weitere Hinweise**	*„Herzlichen Dank an Sie für die interessante Diskussion. Wir beenden an dieser Stelle die Tonbandaufzeichnung.“*	

	Wie geht es mit der Studie weiter? Ergebnisse sind wo und wann einsehbar? Wann treten wir wieder zusammen in Kontakt?<br>Ausfüllen Kurzfragebogen	
**Nachbereitung**	Fotodokumentation Metaplan/Flipchart<br>Sicherung Audiodatei<br>Kurzdokumentation Fokusgruppe<br>Dokumentation Pseudonyme	Kamera<br>Laptop<br>Dokument: Kurzdokumen-tation
*) Zwei vorgelagerte Fragenblöcke zu den Erwartungen und Anforderungen der Teilnehmenden an die Gestaltung der Versorgung auf der NLU sind nicht Gegenstand dieser hier berichteten Forschung und darum hier nicht enthalten.		

Übersicht Kurzfragebogen

Bitte beantworten Sie uns folgende Fragen zu Ihrer Person und Ihrem Arbeitsplatz, damit wir Aussagen über die Zusammensetzung und Repräsentativität der Gruppeninterviews treffen können. Ihre Angaben werden ausschließlich anonymisiert verwendet.

Supplementary Table 6: German Version: Short Questionnaire

**1. Wie alt sind Sie?**	
☐ unter 25 Jahre	☐ 25 bis unter 35 Jahre
☐ 35 bis unter 45 Jahre	☐ 45 bis unter 55 Jahre
☐ 55 bis unter 65 Jahre	☐ 65 Jahre und darüber
**2. Wie lange arbeiten Sie schon im Gesundheitswesen (ohne Ausbildung)?**	
☐ Ich bin noch in der Ausbildung	☐ 6 bis 10 Jahre
☐ weniger als 1 Jahr	☐ 11 bis 20 Jahre
☐ 1 bis 5 Jahre	☐ mehr als 20 Jahre
**3. Mit welcher höchsten (Berufs-)Qualifikation sind sie aktuell tätig?**	
☐ 3-jährige Berufsausbildung ohne akademischen Abschluss	
☐ B.A. / B.Sc.	
☐ Diplom/ M.A. / M.Sc.	
☐ Staatsexamen Medizin	
☐ Promotion	
☐ eine andere Qualifikation: ____________________	
**4. Welcher Arbeitsschwerpunkt entspricht am ehesten Ihrer Tätigkeit?**	
☐ direkte pflegerische Versorgung	
☐ Pflegemanagement	
☐ Medizin	
☐ Therapieberufe (Ergo-, Physio-, Logopädie und andere)	
☐ ein anderer Arbeitsschwerpunkt: ____________________	
**5. Wie lange arbeiten Sie schon im Klinikum Bremerhaven Reinkenheide?**	
☐ weniger als 1 Jahr	☐ 11 bis 20 Jahre
☐ 1 bis 5 Jahre	☐ mehr als 20 Jahre
☐ 6 bis 10 Jahre	
**6. Welchem Fachbereich ordnen Sie sich zu?**	
____________________	
**7. Welchem Geschlecht ordnen Sie sich zu?**	
☐ weiblich ☐ männlich ☐ divers	
