# Supplementary figures and images for: Initial programme theory developing for interprofessional case discussions (InCaD) in acute hospital care: a realist approach
Source: BMC Health Serv Res. 2025 Dec 11;26:21. doi: 10.1186/s12913-025-13865-5 (PMC12771898; doi:10.1186/s12913-025-13865-5)

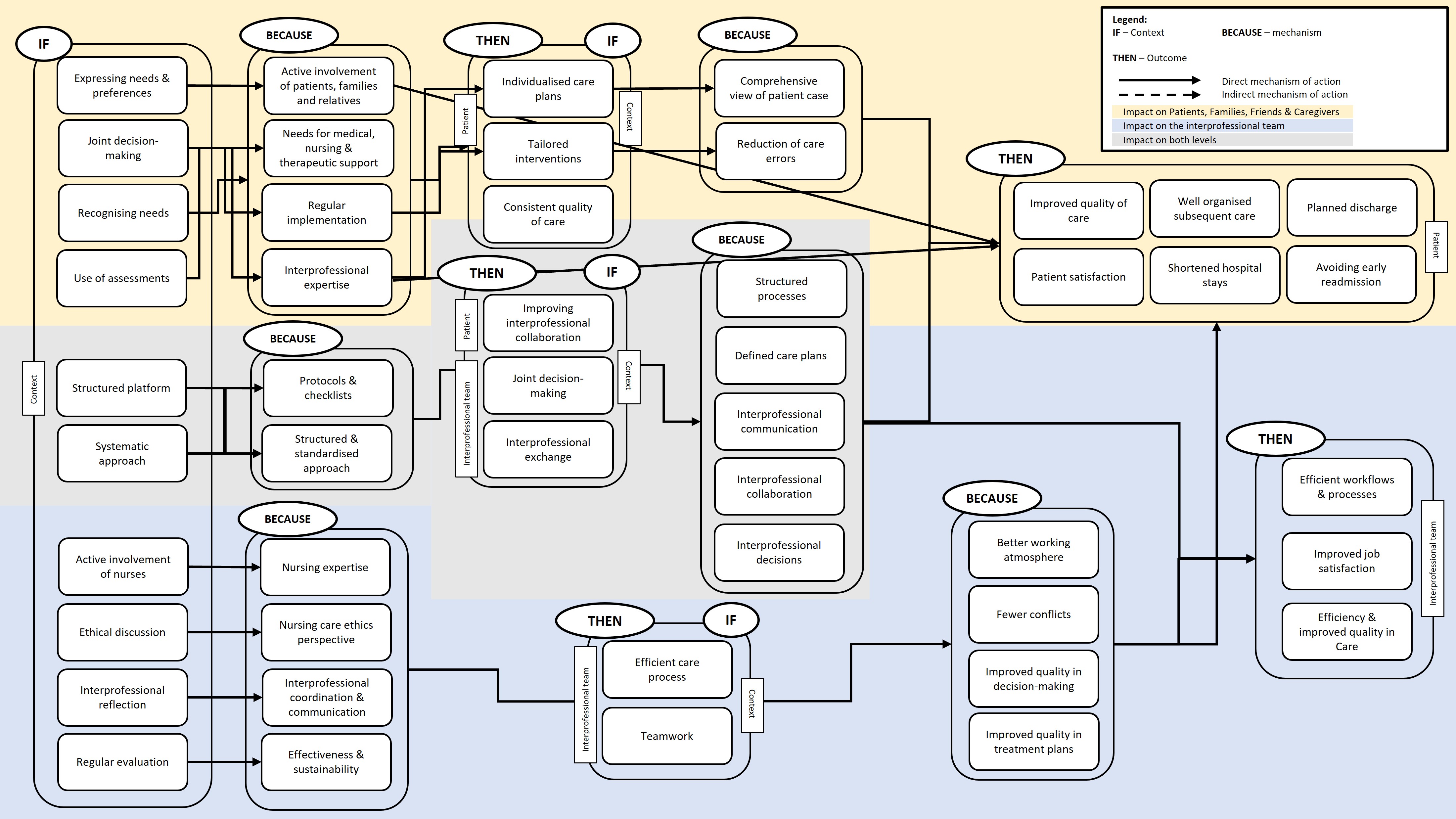

Supplement: Supplementary file 4 — Supplementary Material 4 [file 12913_2025_13865_MOESM4_ESM.jpg]

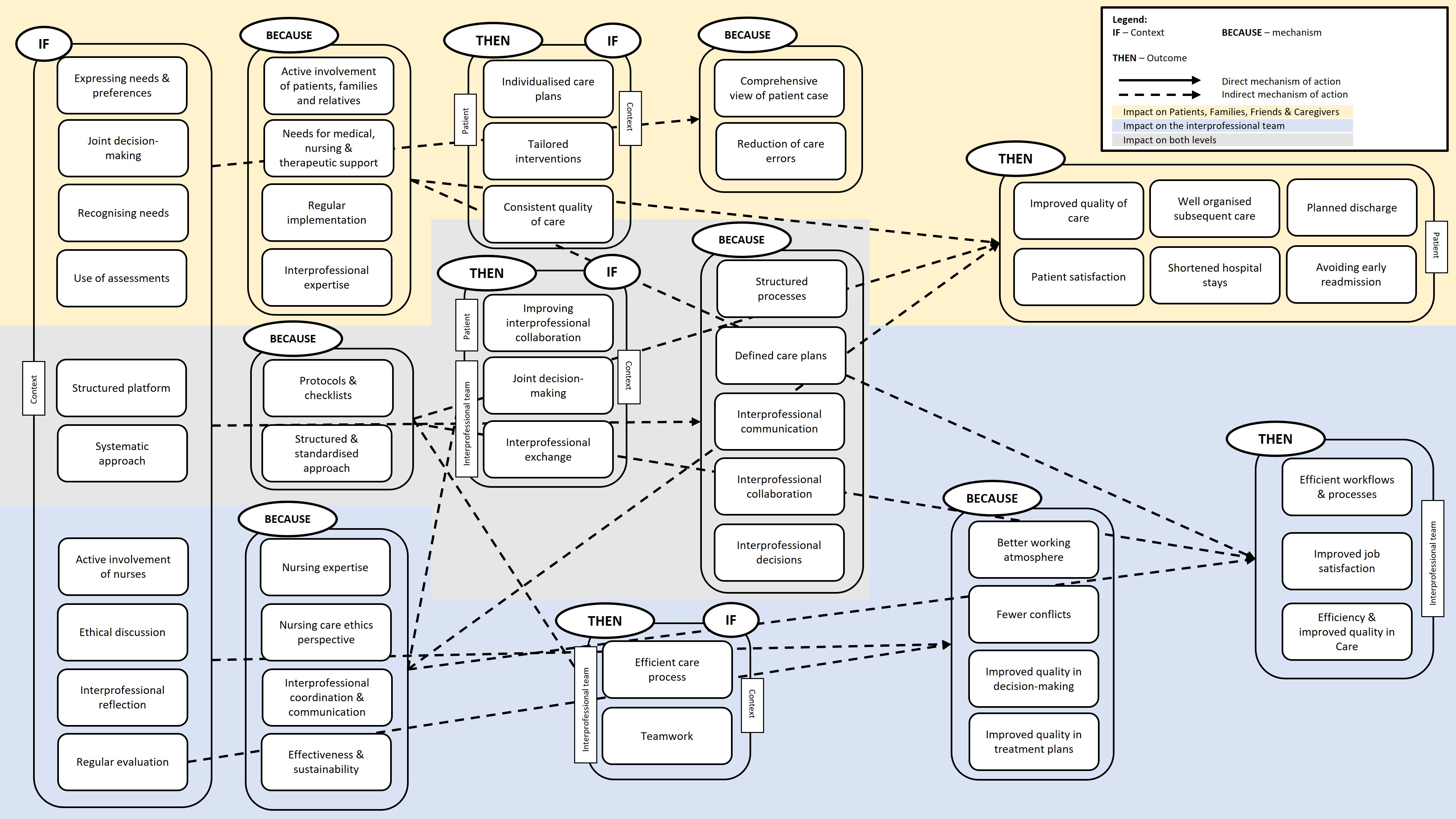

Supplement: Supplementary file 5 — Supplementary Material 5 [file 12913_2025_13865_MOESM5_ESM.jpg]
